# Knowledge and attitude of nosocomial infection prevention and control precautions among healthcare personnel at Kiruddu Referral Hospital in Kampala, Uganda

**DOI:** 10.1186/s12913-025-12219-5

**Published:** 2025-01-28

**Authors:** Newton Ekakoro, Ritah Nakayinga, Martha A. Kaddumukasa, Maria Mbatudde

**Affiliations:** Department of Biological Sciences, Faculty of Science, Kyambogo University, Kampala, Uganda

**Keywords:** Attitudes, COVID-19, Health workers, Infection prevention, Knowledge, Perceptions, SARS-CoV-2

## Abstract

**Background:**

A key concern for global public health is nosocomial infections. Essential to the fight against nosocomial infection, is healthcare professionals’ knowledge and attitudes. Therefore, this study investigated healthcare professionals’ knowledge and attitudes toward nosocomial infection at the Kiruddu Referral Hospital, Kampala, Uganda.

**Methods:**

A facility-based cross-sectional study was carried out at Kiruddu Referral Hospital in Kampala, Uganda. We selected the participants using simple random sampling. Data were collected from a total of 78 healthcare personnel using pretested, structured, self-administered questionnaires. We used SPSS version 20.0 for data analysis and applied descriptive statistics to present the frequencies and percentages. Pearson’s Chi-square test was used to evaluate the association between independent factors and knowledge and attitude (KA) ratings on hospital-acquired infection (HAI) prevention. P-values less than 0.05 were regarded as statistically significant.

**Results:**

Among the different categories of health workers, doctors exhibited the highest level of knowledge. There was a significant association between knowledge scores and occupation (χ^2^LR = 25.610; *P* = 0.000). The mean knowledge scores across different infection prevention aspects were as follows: hand hygiene (82.2 ± 18.9), PPE use (71.8 ± 23.1), sharp disposal and sharp injuries (59.2 ± 25.7), and waste management (57.4 ± 29.9). Notably, 20.5% of participants did not change PPE between patients, and 44.9% indicated that their workload negatively impacted their ability to follow infection prevention standards.

**Conclusion:**

The study highlighted gaps in healthcare personnel’s knowledge and attitudes toward infection prevention. It is therefore important to provide regular targeted training programs emphasizing underrepresented areas, PPE availability, strengthen policy enforcement, and integrate infection prevention education into medical and nursing curricula.

**Supplementary Information:**

The online version contains supplementary material available at 10.1186/s12913-025-12219-5.

## Introduction

Globally, infections continue to pose a significant burden on healthcare service delivery, contributing to increased healthcare costs and creating setbacks in achieving optimal health outcomes [1]. Infection Prevention and Control (IPC) is a practical and evidence – based approach aimed at minimizing the occurrence of avoidable infections that can harm both patients and healthcare professionals [2]. Adhering to standard IPC protocols is essential to prevent and reduce the risk of infectious disease transmission among patients, healthcare personnel, and visitors in health facilities [3]. In addition to safeguarding patient safety, effective IP practices enhance universal health coverage standards [4]. Healthcare personnel are frequently exposed to infectious body fluids, blood, and body parts, which can lead to serious or even fatal illnesses [5, 6].

Hospital-acquired infections (HAIs), sometimes referred to as nosocomial infections, are infections that occur during medical treatment but are not present at the time of patient admission [6, 7]. These infections typically manifest at least 48 to 72 h after hospital admission or up to 10 days post-discharge [8, 9]. The incidence of nosocomial infections is increasing globally, posing a significant challenge to healthcare systems [10, 11]. The prevalence of HAIs varies significantly across regions, ranging from 5.7% to 19.2% in low-income countries, compared to 7.5% in high-income countries [12]. Nosocomial infection rates range from less than 1% in various European and American nations to over 40% in regions of Asia, Latin America, and Sub-Saharan Africa [6]. In Africa, it is estimated that between 3 and 15% of hospitalized patients develop HAIs, with a range of 1.6% to 28.7% reported specifically in Sub-Saharan Africa [13, 14]. In Uganda the prevalence of HAIs is 34%, with the majority of cases involving multiple infections [15].

Patients, healthcare providers, and communities are at serious danger when Infection Prevention (IP) measures are not carried out correctly [10]. All medical personnel must follow IP practices in order to safeguard their own health, lower the risk of nosocomial infections, and improve patient safety [16, 17]. Healthcare personnel face constant exposure to infectious agents from patients, but these risks can be significantly reduced by strictly following IP protocols [18]. The effectiveness of IP measures largely depends on healthcare personnel knowledge, and attitudes [19].

Despite widespread awareness of infection prevention and control (IPC) measures, studies reveal significant gaps in adherence among healthcare personnel, particularly in low- and middle-income countries. Allegranzi et al. reported suboptimal hand hygiene compliance [20], while World Health Organization identified challenges in proper PPE selection and disposal [21]. Adebayo et al. highlighted deficiencies in sharp disposal, increasing risks of bloodborne infections [22]. Variability in IPC adherence exists across occupational categories, with doctors excelling in theoretical knowledge and nurses showing better practical compliance [23]. Limited access to structured IPC training and refresher courses, as observed in Ethiopia and Uganda, exacerbates the problem [24]. High workloads, resource constraints, and poor institutional support further hinder IPC compliance [25]. This study comprehensively evaluates healthcare personnel’s IPC knowledge and attitudes.

Evaluating healthcare personnel’ existing Knowledge and Attitudes (KA) on infection control is an essential first step in creating and executing a successful program [26]. Delivering high-quality healthcare relies on an understanding of evidence-based infection prevention guidelines [6]. Non-compliance with these guidelines, often due to a lack of awareness or understanding among nurses and other healthcare staff, can lead to increased risk of nosocomial infections [27]. By providing a detailed assessment of knowledge and attitudes, this study aimed to inform targeted interventions to improve infection prevention precautions among healthcare personnel. The findings will have the potential to enhance patient safety, reduce the burden of nosocomial infections, and contribute to the development of sustainable infection control strategies in healthcare settings.

## Methods

### Study design

A cross-sectional study using a quantitative approach was carried out at a healthcare facility to assess knowledge and attitudes toward nosocomial infection prevention and control measures. This approach aligns with similar studies conducted in Ethiopia [4], South Africa [28], Uganda [29], and China [30].

### Study setting

**Fig. 1:**
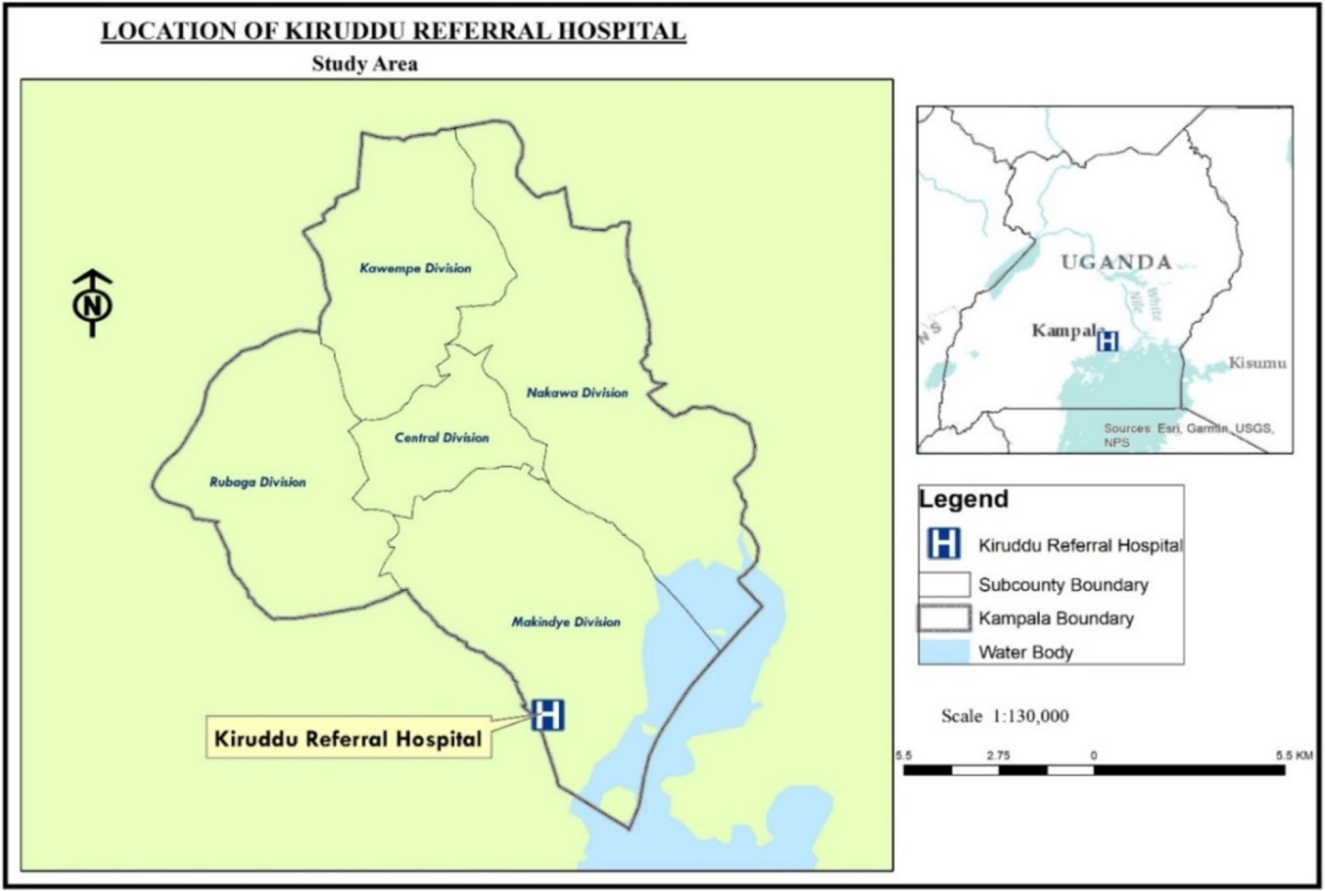
Study area

This study was conducted at Kiruddu Referral Hospital situated in Makindye Division, one of five divisions in Kampala, Uganda’s capital city: Coordinates: 0°14′53.0″N, 32°36′45.0″E). The hospital a tertiary referral facility was selected because of its role in offering specialized healthcare services to a large and diverse population referred from lower-level facilities across the Central Region and beyond. This includes critically ill patients who are particularly susceptible to healthcare-associated infections (HAIs) [31]. As part of Uganda’s public healthcare system under the Ministry of Health, the hospital provides general medicine, surgery, intensive care, and specialized outpatient services. With its multidisciplinary healthcare personnel and high patient volumes, Kiruddu offers an ideal setting for assessing knowledge and attitudes toward nosocomial infection prevention and control. The hospital faces challenges such as limited resources, overcrowding and inadequate infection control measures [32, 33], which contribute to a high burden of HAIs, including surgical site infections, bloodstream infections and catheter-associated urinary tract infections [34, 35]. This study aimed to identify gaps in infection prevention knowledge and attitudes to help inform targeted interventions that enhance infection control practices, patient safety and healthcare outcomes at Kiruddu and similar facilities (Fig. 1).

### Study population

The target population for this study consisted of 130 healthcare personnel working at Kiruddu Referral Hospital This group represents a diverse range of healthcare cadres, responsible for delivering direct care. Their roles and responsibilities directly influence the prevention and control of nosocomial infections (HAIs). The healthcare personnel that were included in the study were doctors, nurses, pharmacists, resident physicians and laboratory personnel. The inclusion of various cadres of healthcare personnel was intentional to provide a holistic assessment of knowledge and attitudes toward nosocomial infection prevention.

### Sample size determination and sampling criteria

The Fisher’s formula for estimating single proportions [36] was used to determine the optimal sample size based on the probability of selecting a specific choice, the desired confidence level, and the acceptable margin of error. This formula is particularly effective for large populations. When the total sample size was less than 10,000, a correction formula was applied to estimate the minimum required sample size [37].

Fisher’s formulae

$$n=\boldsymbol{ }\frac{({{{\varvec{Z}}}_{{}^{\propto }\!\left/ \!{}_{2}\right.})}^{2}{\varvec{p}}{\varvec{q}}}{{{\varvec{d}}}^{2}}= \frac{3.8416 \times 0.203 \times 0.797}{0.0025}= \frac{0.6216}{0.0025}= 248.64= 249\text{ participants}$$

Here, n = minimum required sample size in population greater than 10,000, Z_α/2_ = the standard normal deviation which was set at a 95% confidence level = 1.96, P = prevalence of knowledge of healthcare personnel at 20.3% [38] with the allowable margin of error of 5% (d = 0.05) and q = 1 − p.

Correction formulae

This was used since the source population was less than 10,000.

$${n}_{f}=\boldsymbol{ }\frac{n}{1+ {}^{n}\!\left/ \!{}_{N}\right.}=\boldsymbol{ }\frac{249}{1+ {}^{249}\!\left/ \!{}_{130}\right.}=\boldsymbol{ }\frac{249}{2.915}=\boldsymbol{ }85\ participants$$

Here, n = minimum required sample size in population less than 10,000, N = population size at the facility, n_f_ = corrected sample size.

The minimum sample size was raised by 5% to accommodate for possible non-responses, incomplete, or missing questionnaires. This resulted in a final sample size of 89.3, which was rounded down to 89 participants. Accordingly, 89 questionnaires were distributed to potential participants, who were selected using a simple random sampling method, as employed in several similar studies [39–41].

### Sampling procedure

A list of healthcare personnel from various wards was compiled to identify eligible participants for the study. This list, which included all potential members of the target population, served as the sampling frame. Participants were then randomly selected, through the use of a random number generator.

### Variables of the study

The dependent variables emphasized the extent of knowledge regarding infection prevention measures and attitudes toward adherence to these measures. Knowledge was evaluated in several domains: the general concept of nosocomial infections, hand hygiene practices, utilization of Personal Protective Equipment (PPE), the disposal of sharps and management of sharp injuries, as well as waste management. Attitudes were classified as either positive or negative, reflecting healthcare personnel’s overall perception and willingness to comply with infection control measures. The independent variables concentrated on the occupational categories (doctor, nurse, resident physician, pharmacist, lab technician).

### Study instrument

Structured questionnaires, similar to those used in previous studies [29, 42], were adopted from existing literature on healthcare personnel’ knowledge and attitudes toward Infection Control (IC) and Standard Precautions (SPs) [43, 44]. These questionnaires were modified by the researchers and reviewed by experts for relevance and clarity. The instrument consisted of three sections: Section A covered the socio-demographic characteristics of participants (e.g., age, gender, educational status, IPC training, occupation, and work experience). Section B focused on participants’ knowledge of IPC precautions, containing 35 questions divided into five subcategories: general concepts (6 questions), hand hygiene (8 questions), PPE use (10 questions), sharp disposal (6 questions), and waste management (5 questions). Each item in this section was scored using a true/false format. Section C assessed participants’ attitudes toward IPC, with 13 questions rated on a 3-point Likert scale with (disagree, not sure and agree).

The knowledge and attitude scores for each participant were then used to calculate percentage scores. Knowledge levels were categorized as good (> 80%), moderate (50%—80%), and poor (< 50%). Attitude levels were classified as positive (> 60%) or negative (< 60%).

A preliminary investigation was carried out with ten medical staff members from the hospital in order to strengthen the validity of the surveys. The pilot data were not included in the final analysis; instead, the pilot results were utilized to improve the data gathering tool’s logic and consistency.

### Validity and reliability

A preliminary investigation was conducted involving ten medical staff members, comprising two representatives each from doctors, nurses, pharmacists, resident physicians, and laboratory personnel at the hospital in order to strengthen the validity of the surveys. The selection of ten participants was guided by the need to provide a balance between feasibility, time efficiency, and the diversity required to capture insights across different healthcare roles [45]. The pilot data were not included in the final analysis; instead, the pilot results were utilized to improve the data gathering tool’s logic and consistency. However, the reliability of the tool was assessed using Cronbach’s alpha (α) test and yielded a reliability coefficient of 0.97 for both Section B and Section C, which was deemed acceptable.

### Data collection

The questionnaires were distributed throughout the three weeks period. Healthcare personnel from the hospital were invited to complete them during their lunch breaks and off-duty hours. They were required to return the completed questionnaires, and follow-ups were conducted in various wards to ensure their collection. Out of the 89 questionnaires distributed, 78 were completed, resulting in a response rate of 87.6%. These completed questionnaires were used in the final analysis.

### Data analysis

The researchers validated and cleaned the gathered data to ensure correctness and completeness. IBM SPSS software Version 20.0 was used to code, input, and analyse the data. The codes for knowledge questions were '1' for right answers and '0' for wrong responses. Attitudes were measured using a 3 – point Likert scale with responses coded as 3 for “agree,” 2 for “not sure,” and 1 for “disagree.” The data was found to be normally distributed, as confirmed by the Shapiro–Wilk test, and therefore, the mean scores were used for analysis.The socio demographic characteristics, knowledge scores and attitude scores for each healthcare personnel were presented in tabular form, with frequencies and percentages for categorical variables and as measure of central tendency (mean) and dispersion (standard deviation) for continuous variables. Chi – square test statistics were utilized for bivariate analysis in order to investigate the associations between the variables. Statistical significance was defined as a two-tailed significance level of 5%.

### Ethics approval and consent to participate

Clarke International University granted ethical approval for the study under ethical code CLARKE – 2022 – 342. The Declaration of Helsinki’s guiding principles were followed in the conduct of the study. Every procedure was completed in accordance with the applicable laws and manuals. Before the study started, written informed consent was obtained from each participant. The consent form furnished participants with comprehensive details regarding the aim, importance, and methodology of the study. Participants received guarantees that their participation was optional, their queries would be answered, and their information would be kept private. Additionally, participants were told that they could leave the study at any time.

## Results

### Characteristics of the healthcare personnel that participated in the study

A total of 78 healthcare personnel were included in the study. The majority of participants were aged between 21 and 29 years (64.1%), with a higher proportion of males (64.1%) compared to females (35.9%). Most participants (73.1%) held a bachelor’s degree, and approximately two-thirds had 1 to 5 years of work experience. Notably, 16.0% of doctors, 16.7% of nurses, 25.0% of resident physicians, and 40.0% of pharmacists had not received previous training in infection prevention and control. A substantial proportion of participants (80.8%) demonstrated a moderate level of knowledge, while 66.7% had a positive attitude towards nosocomial infection prevention, with the majority being laboratory technicians. The study found a statistically significant association between work categories and knowledge (χ^2^ = 25.610, *P* = 0.000) (Table 1).

**Table 1:** Characteristics of the healthcare personnel

	**Healthcare personnel, n (%)**					
	**Doctor (*N* = 25)**	**Nurse (*N* = 30)**	**R. Physician (*N* = 8)**	**Pharmacist (*N* = 5)**	**Lab tech (*N* = 10)**	**Total (*N* = 78)**
**Gender**						
Female	5 (20.0)	16 (53.3)	3 (37.5)	2 (40.0)	2 (20.0)	28 (35.9)
Male	20 (80.0)	14 (46.7)	5 (62.5)	3 (60.0)	8 (80.0)	50 (64.1)
**Age**						
21—29 years	20 (80.0)	19 (63.3)	4 (50.0)	4 (80.0)	3 (30.0)	50 (64.1)
30—39 years	4 (16.0)	7 (23.3)	4 (50.0)	1 (20.0)	2 (20.0)	18 (23.1)
40—49 years	1 (4.0)	3 (10.0)	0 (0.0)	0 (0.0)	2 (20.0)	6 (7.7)
> = 50 years	0 (0.0)	1 (3.3)	0 (0.0)	0 (0.0)	3 (30.0)	4 (5.1)
**Marital status**						
Single	21 (84.0)	18 (60.0)	3 (37.5)	3 (60.0)	2 (20.0)	47 (60.3)
Married	4 (16.0)	11 (36.7)	5 (62.5)	2 (40.0)	7 (70.0)	29 (37.2)
Widowed	0 (0.0)	1 (3.3)	0 (0.0)	0 (0.0)	1 (10.0)	2 (2.6)
**Education level**						
Diploma	0 (0.0)	13 (43.3)	3 (37.5)	0 (0.0)	3 (30.0)	19 (24.4)
Bachelor’s	25 (100.0)	17 (56.7)	5 (62.5)	5 (100.0)	5 (50.0)	57 (73.1)
Master’s	0 (0.0)	0 (0.0)	0 (0.0)	0 (0.0)	2 (20.0)	2 (2.6)
**Work experience**						
Score, Mean ± SD	1.16 ± 0.473	1.87 ± 1.332	1.50 ± 0.535	1.20 ± 0.447	3.00 ± 1.491	1.71 ± 1.175
1—5 years	22 (88.0)	19 (63.3)	4 (50.0)	4 (80.0)	2 (20.0)	51 (65.4)
6—10 years	2 (8.0)	2 (6.7)	4 (50.0)	1 (20.0)	2 (20.0)	11 (14.1)
11—15 years	1 (4.0)	6 (20.0)	0 (0.0)	0 (0.0)	2 (20.0)	9 (11.5)
16—20 years	0 (0.0)	0 (0.0)	0 (0.0)	0 (0.0)	2 (20.0)	2 (2.6)
> 20 years	0 (0.0)	3 (10.0)	0 (0.0)	0 (0.0)	2 (20.0)	5 (6.4)
**Shift duration**						
< 8 h	8 (32.0)	8 (26.7)	2 (25.0)	0 (0.0)	3 (30.0)	21 (26.9)
8 h	4 (16.0)	12 (40.0)	4 (50.0)	0 (0.0)	5 (50.0)	25 (32.1)
> 8 h	13 (52.0)	10 (33.3)	2 (25.0)	5 (100.0)	2 (20.0)	32 (41.0)
**Patients per shift**						
Score, Mean ± SD	2.76 ± 1.234	3.57 ± 1.251	4.13 ± 0.835	5.00 ± 0.000	4.60 ± 0.699	3.59 ± 1.304
< 5	3 (12.0)	0 (0.0)	0 (0.0)	0 (0.0)	0 (0.0)	3 (3.8)
5—10	9 (36.0)	8 (26.7)	0 (0.0)	0 (0.0)	0 (0.0)	17 (21.8)
11—15	8 (32.0)	8 (26.7)	2 (25.0)	0 (0.0)	1 (10.0)	19 (24.4)
16—20	1 (4.0)	3 (10.0)	3 (37.5)	0 (0.0)	2 (20.0)	9 (11.5)
> 20	4 (16.0)	11 (36.7)	3 (37.5)	5 (100.0)	7 (70.0)	30 (38.5)
**OSH training**						
No	9 (36.0)	10 (33.3)	4 (50.0)	0 (0.0)	0 (0.0)	23 (29.5)
Yes	16 (64.0)	20 (66.7)	4 (50.0)	5 (100.0)	10 (100.0)	55 (70.5)
**IPC training**						
No	4 (16.0)	5 (16.7)	2 (25.0)	2 (40.0)	0 (0.0)	13 (16.7)
Yes	21 (84.0)	25 (83.3)	6 (75.0)	3 (60.0)	10 (100.0)	65 (83.3)
**Knowledge**						
Score, Mean ± SD	1.76 ± 0.436	1.90 ± 0.305	2.50 ± 0.535	2.00 ± 0.000	1.80 ± 0.422	1.91 ± 0.432
Good	6 (24.0)	3 (10.0)	0 (0.0)	0 (0.0)	2 (20.0)	11 (14.1)
Moderate	19 (76.0)	27 (90.0)	4 (50.0)	5 (100.0)	8 (80.0)	63 (80.8)
Poor	0 (0.0)	0 (0.0)	4 (50.0)	0 (0.0)	0 (0.0)	4 (5.1)
	χ^2^_LR_ = 25.610; df = 8; *P* = 0.000^*^					
**Attitude**						
Score, Mean ± SD	1.32 ± 0.476	1.33 ± 0.479	1.50 ± 0.535	1.40 ± 0.548	1.20 ± 0.422	1.33 ± 0.474
Positive	17 (68.0)	20 (66.7)	4 (50.0)	3 (60.0)	8 (80.0)	52 (66.7)
Negative	8 (32.0)	10 (33.3)	4 (50.0)	2 (40.0)	2 (20.0)	26 (33.3)
	χ^2^_LR_ = 1.933; df = 4; *P* = 0.7					

*SD* Standard Deviation, *OSH* Occupational Safety and Health, *IPC* Infection Prevention and Control, *R. physician* Resident physician, *Lab tech* Laboratory technician, *(χ*^*2*^_*LR*_*)* Chi-square, Likelihood Ratio, *df* degree of freedom, *P* P – value

^*^Significant if *P* < 0.05

### General concept of nosocomial infections

Doctors demonstrated higher knowledge compared to other healthcare personnel, with a mean percentage score of 80.0 ± 11.31. Participants generally showed lower knowledge about the duration it takes for nosocomial infections to appear. Pharmacists had the lowest level of knowledge regarding the causes of nosocomial infections. Over 84.0% of doctors, 90.0% of nurses, 75.0% of physicians, and 70.0% of laboratory technicians correctly identified that all patients can be sources of infection regardless of their diagnoses. However, overall knowledge about the sources of infection was found to be limited among participants (Table 2).

**Table 2:** Distribution of health personnel correct responses about general concepts of nosocomial infections

	**Healthcare personnel, n (%)**					
**Items**	**Doctor (*N* = 25)**	**Nurse (*N* = 30)**	**Physician (*N* = 8)**	**Pharmacist (*N* = 5)**	**Lab tech (*N* = 10)**	**Total (*N* = 78)**
Infections contracted at a hospital are known as nosocomial infections **(True)**	23 (92.0)	30 (100.0)	5 (62.5)	4 (80.0)	10 (100.0)	72 (92.3)
An infection is nosocomial if it appears after;(48 – 72 h) **(True)**	17 (68.0)	12 (40.0)	1 (12.5)	4 (80.0)	4 (40.0)	38 (48.7)
Medical equipment that is infected might spread nosocomial diseases **(True)**	22 (88.0)	26 (86.7)	7 (87.5)	4 (80.0)	10 (100.0)	69 (88.5)
Nosocomial infection can be caused by bacteria only found in and around the hospital **(True)**	21 (84.0)	23 (76.7)	4 (50.0)	2 (40.0)	6 (60.0)	56 (71.8)
Regardless of their diagnosis, any patient can spread infection **(True)**	21 (84.0)	27 (90.0)	6 (75.0)	3 (60.0)	7 (70.0)	64 (82.1)
All bodily fluids aside from sweat should be considered potential infection sources **(True)**	16 (64.0)	12 (40.0)	3 (37.5)	0 (0.0)	3 (30.0)	34 (43.6)
**Mean ± SD (Percentage score)**	**80.0 ± 11.31**	**72.2 ± 26.05**	**54.2 ± 27.00**	**56.7 ± 32.04**	**66.7 ± 29.44**	**71.2 ± 20.65**

### Hand hygiene and personal protective equipment use knowledge

With a mean score of 88.8 ± 16.4, laboratory technicians showed the highest level of knowledge regarding hand hygiene. More than 92.3 percent of participants said that the best way to avoid nosocomial infections is to practice good hand hygiene. But fewer than half (44.9%) knew that it was important to wash your hands before handling patients who had respiratory illnesses. The majority, laboratory technologists (90.0%), knew the standard period for hand washing better than seventy-five percent (79.5%). With a mean score of 77.6 ± 21.3, doctors were found to have the highest degree of knowledge on the usage of personal protective equipment (PPE) in the study. Only 34.6% of participants recognized that PPE is essential not only for laboratory and cleaning staff but also for overall safety. Less than half (46.2%) correctly identified that used PPE should not be disposed of in trash containers. Additionally, over 69.2% of participants correctly understood that masks and gloves should not be reused when dealing with the same patient (Table 3).

**Table 3:** Distribution of health personnel correct responses about Hand hygiene and personal protective equipment use

	**Healthcare personnel, n (%)**					
**Items**	**Doctors (*N* = 25)**	**Nurses (*N* = 30)**	**Physician (*N* = 8)**	**Pharmacist (*N* = 5)**	**Lab tech (*N* = 10)**	**Total (*N* = 78)**
**Hand hygiene**						
Practicing good hand cleanliness is the best defense against nosocomial infections **(True)**	25 (100.0)	28 (93.3)	5 (62.5)	4 (80.0)	10 (100.0)	72 (92.3)
Those who have respiratory illnesses need to practice good hand hygiene **(True)**	11 (44.0)	12 (40.0)	5 (62.5)	2 (40.0)	5 (50.0)	35 (44.9)
The danger of spreading hospital acquired germs is reduced by washing hands with soap and water **(True)**	24 (96.0)	30 (100.0)	6 (75.0)	5 (100.0)	9 (90.0)	74 (94.9)
After taking off your gloves, you should wash your hands **(True)**	24 (96.0)	30 (100.0)	6 (75.0)	5 (100.0)	9 (90.0)	74 (94.9)
Using an alcohol-based antiseptic for hand care is equally as effective as using soap if hands are not dirty **(True)**	13 (52.0)	20 (66.7)	3 (37.5)	4 (80.0)	9 (90.0)	49 (62.8)
Putting on gloves makes washing your hands unnecessary **(False)**	23 (92.0)	30 (100.0)	5 (62.5)	5 (100.0)	10 (100.0)	73 (93.6)
Before and after having direct patient contact, hand hygiene should be practised **(True)**	22 (88.0)	30 (100.0)	7 (87.5)	5 (100.0)	10 (100.0)	74 (94.9)
The recommended minimum time for normal hand washing is between 40 and 60 s **(True)**	22 (88.0)	23 (76.7)	6 (75.0)	2 (40.0)	9 (90.0)	62 (79.5)
**Mean ± SD (Percentage score)**	**82.0 ± 21.5**	**84.6 ± 22.0**	**67.2 ± 14.8**	**80.0 ± 26.2**	**88.8 ± 16.4**	**82.2 ± 18.9**
**Personal Protective Equipment (PPE) use**						
If there is no obvious contamination on the gloves, the same pair can be used for several patients **(False)**	23 (92.0)	28 (93.3)	5 (62.5)	4 (80.0)	10 (100.0)	70 (89.7)
Protective barriers against infection are provided by PPEs such as masks and head coverings **(True)**	24 (96.0)	29 (96.7)	5 (62.5)	4 (80.0)	10 (100.0)	72 (92.3)
The danger of developing nosocomial infections is eliminated by the use of PPEs **(True)**	23 (92.0)	24 (80.0)	6 (75.0)	4 (80.0)	10 (100.0)	67 (85.9)
For their safety, PPEs are only appropriate for laboratory and cleaning employees **(False)**	11 (44.0)	11 (36.7)	1 (12.5)	2 (40.0)	2 (20.0)	27 (34.6)
Only when there is blood contact should PPEs be worn **(False)**	21 (84.0)	26 (86.7)	4 (50.0)	4 (80.0)	9 (90.0)	64 (82.1)
After being cleaned properly, gloves and masks can be used again **(False)**	24 (96.0)	30 (100.0)	5 (62.5)	5 (100.0)	10 (100.0)	74 (94.9)
Old PPE should be disposed of in standard trash containers **(False)**	16 (64.0)	11 (36.7)	2 (25.0)	2 (40.0)	5 (50.0)	36 (46.2)
Gloves should be changed when doing different procedures on the same patient **(True)**	23 (92.0)	27 (90.0)	4 (50.0)	3 (60.0)	8 (80.0)	65 (83.3)
The most protective masks are those composed of cotton or gauze **(False)**	10 (40.0)	12 (40.0)	2 (25.0)	2 (40.0)	5 (50.0)	31 (39.7)
If working with the same thing, masks and gloves can be reused **(False)**	19 (76.0)	19 (63.3)	4 (50.0)	4 (80.0)	8 (80.0)	54 (69.2)
**Mean ± SD (Percentage score)**	**77.6 ± 21.3**	**72.3 ± 25.9**	**47.5 ± 20.2**	**68.0 ± 21.5**	**77.0 ± 27.9**	**71.8 ± 23.1**

### Sharp disposal and sharp injuries and waste management

Laboratory technicians exhibited the highest level of knowledge among all healthcare personnel, with a mean score of 70.0 ± 27.6. Only 52.6% of participants correctly identified that used needles should not be recapped. More than half (69.2%) of participants accurately stated that used needles shouldn’t be bent, and 83.3% correctly stated that HIV-positive patients’ unintentional sharp injuries are treated with post-exposure prophylaxis. But just 12.8% were aware that shredded sharps should be disposed of after being contaminated. In terms of waste management, laboratory technicians again showed the highest knowledge, with a mean score of 64.0 ± 34.4. Only 19.2% of participants correctly noted that hospital wards should be cleaned twice within a 24-h period, and less than half (35.9%) knew that used PPE should not be discarded through regular municipal waste disposal systems (Table 4).

**Table 4:** Distribution of health personnel correct responses about sharp disposal and sharp injuries and waste management

	**Healthcare personnel, n (%)**					
**Items**	**Doctors (*N* = 25)**	**Nurses (*N* = 30)**	**Physician (*N* = 8)**	**Pharmacist (*N* = 5)**	**Lab tech (*N* = 10)**	**Total (*N* = 78)**
**Sharp disposal and sharp injuries**						
To avoid injury, used needles should be recapped after use **(False)**	11 (44.0)	22 (73.3)	1 (12.5)	0 (0.0)	7 (70.0)	41 (52.6)
After use, used needles should be twisted to prevent injury **(False)**	16 (64.0)	24 (80.0)	1 (12.5)	4 (80.0)	9 (90.0)	54 (69.2)
When disposing of soiled sharps, shred them first **(True)**	3 (12.0)	3 (10.0)	2 (25.0)	0 (0.0)	2 (20.0)	10 (12.8)
Sharps injuries ought to be treated without requiring reporting **(False)**	18 (72.0)	28 (93.3)	4 (50.0)	5 (100.0)	7 (70.0)	62 (79.5)
In regular practice, needle-stick injuries are the least common **(False)**	13 (52.0)	19 (63.3)	3 (37.5)	3 (60.0)	7 (70.0)	45 (57.7)
Injuries from an HIV-positive patient are managed with post-exposure prophylaxis **(True)**	21 (84.0)	27 (90.0)	4 (50.0)	3 (60.0)	10 (100.0)	65 (83.3)
**Mean ± SD (Percentage score)**	**54.7 ± 25.3**	**68.3 ± 30.6**	**31.3 ± 17.2**	**50.0 ± 41.5**	**70.0 ± 27.6**	**59.2 ± 25.7**
**Waste management**						
Hospital waste has to be sorted before disposal **(True)**	23 (92.0)	28 (93.3)	5 (62.5)	5 (100.0)	9 (90.0)	70 (89.7)
Cleaning and disinfection are the same **(False)**	20 (80.0)	27 (90.0)	3 (37.5)	5 (100.0)	9 (90.0)	64 (82.1)
Hospital wards have to be cleaned only 2 times in 24 h **(False)**	5 (20.0)	7 (23.3)	1 (12.5)	1 (20.0)	1 (10.0)	15 (19.2)
Waste at the hospital should be collected twice monthly **(False)**	16 (64.0)	17 (56.7)	4 (50.0)	2 (40.0)	8 (80.0)	47 (60.3)
It is necessary to dispose of used PPE using the standard municipal disposal methods **(False)**	12 (48.0)	9 (30.0)	1 (12.5)	1 (20.0)	5 (50.0)	28 (35.9)
**Mean ± SD (Percentage score)**	**60.8 ± 28.2**	**58.7 ± 32.6**	**35.0 ± 22.4**	**56.0 ± 41.0**	**64.0 ± 34.4**	**57.4 ± 29.9**

### Attitude towards nosocomial infection prevention and control

In our study, 29.5% of participants reported that hand sanitizers cause irritation and dryness. A majority, 83.3%, believed that safety boxes should always be placed close to where procedures are performed, and 79.5% confirmed that sharps should be disposed of in designated sharps boxes. Additionally, 80.8% of participants felt that practicing good hand hygiene reduces the risk of patient contamination. However, 20.5% of participants admitted not changing PPE between patients, and 44.9% felt that their ability to adhere to infection prevention standards was affected by their workload. Despite discomfort, 76.9% of participants indicated they would continue to wear essential personal protective equipment. Furthermore, 51.3% preferred alcohol-based hand sanitizers over handwashing with soap and water for feeling safer, and only 21.8% would report for duty even if they had acquired an infection (Table 5).

**Table 5:** Attitude of healthcare personnel towards nosocomial infection prevention and control

Attitude towards nosocomial infection prevention and control	Agree	Not sure	Disagree
	**n (%)**	**n (%)**	**n (%)**
Hand sanitizers irritate and make me feel dry	23 (29.5)	14 (17.9)	41 (52.6)
Safety boxes should always be placed nearby locations where necessary operations are carried out	65 (83.3)	5 (6.4)	8 (10.3)
Usually forget to wash my hands	20 (25.6)	8 (10.3)	50 (64.1)
Sharps should always be disposed in sharps’ boxes	62 (79.5)	6 (7.7)	10 (12.8)
If I practise good hand hygiene am less likely to contaminate my patients	63 (80.8)	8 (10.3)	7 (9.0)
The importance of healthcare personnel in preventing hospital acquired infections is crucial	56 (71.8)	17 (21.8)	5 (6.4)
I would feel uncomfortable telling a healthcare personnel to practise good hand hygiene	26 (33.3)	14 (17.9)	38 (48.7)
I don’t change PPE between patients	16 (20.5)	9 (11.5)	53 (67.9)
Capacity to follow infection prevention standards is impacted by my workload	35 (44.9)	9 (11.5)	34 (43.6)
Despite the discomfort, I would wear the necessary personal protective equipment	60 (76.9)	8 (10.3)	10 (12.8)
Using alcohol-based hand sanitizers makes me feel safer than washing my hands with soap and water	40 (51.3)	10 (12.8)	28 (35.9)
I will still report for duty even though I acquire an infection	17 (21.8)	11 (14.1)	50 (64.1)

## Discussion

Healthcare personnel encounter significant risks of occupational infections, which contribute to morbidity and mortality [46], including *Methicillin-Resistant Staphylococcus aureus* (MRSA), HIV, HBV, HCV and various bacterial and viral infections [40]. Effective infection prevention practices are essential to mitigate these risks; requiring adequate knowledge and positive attitudes among healthcare personnels [47]. Inadequate measures can harm both staff and patients, promoting the spread of nosocomial infections. This study assessed healthcare personnel’s knowledge and attitudes across professional categories to inform strategies for improving infection prevention and control [48].

In our study, only 14.1% of healthcare personnel demonstrated a high level of knowledge about infection prevention. In contrast, a substantial portion (80.8%) exhibited moderate knowledge. This result is lower compared to similar studies, such as those in Nepal 22.0% [49] and Trinidad and Tobago 20.3% [38]. However, it is notable that the proportion of knowledgeable participants in our study is significantly lower than in studies conducted in Saudi Arabia 67.6% [50], Nigeria 51.1% [17], and Ethiopia, with varying proportions reported 71.9% [6]; 59.7% [4]; 70.8% [19]; 90.0% [9].

Discrepancies in IPC knowledge levels between our study and those conducted elsewhere can be attributed to differences in healthcare training, resource availability and cultural and institutional priorities. Countries with well-established training programs, better access to IPC resources such as PPE and guidelines, and structured healthcare systems tend to report higher knowledge levels [51–53]. However, resource-limited settings often face challenges like high workloads and inadequate training, which hinder knowledge acquisition [54].

The findings revealed that 16.7% of participants had not received training on infection prevention, while 29.5% had not received training on occupational health and safety. Studies conducted in Ethiopia and Nepal showed a positive correlation between infection prevention knowledge and training among healthcare personnel [55, 56]. This correlation indicates that updating healthcare personnel’ knowledge of infection prevention principles can enhance their understanding and improve their performance on knowledge assessments [40]. However, some studies have highlighted gaps in training, noting that many healthcare personnel receive no additional training or orientation on infection prevention beyond their initial professional education, or are uncertain about their training status [19, 57]. Furthermore, it has been reported that frontline healthcare personnel often have less access to training compared to administrators [58]. The World Health Organization emphasizes that education and training are fundamental components of effective infection prevention and control programs [48]. These findings highlight the critical need for ongoing training to enhance healthcare personnel’ knowledge and ensure consistent adherence to infection prevention practices.

In our study, doctors significantly outperformed nurses in terms of mean knowledge scores. This finding aligns with a study conducted in Greece, which reported that doctors had higher knowledge scores on SARS-CoV-2 preventive practices compared to nurses [59]. However, this contrast with a survey from Ethiopia that found doctors were less knowledgeable about infection prevention compared to nurses [40]. The discrepancies in knowledge level could be brought by differences in training and professional development opportunities [34], and variations in clinical roles and responsibilities [60].

Participants in our study demonstrated greater knowledge regarding hand hygiene and PPE use compared to other aspects of infection prevention. This could be attributed to continuous training and implementation of institutional practice on hand hygiene and PPE. Healthcare institutions, on the other hand, place a significant emphasis on training concerning these measures, as studies indicate that they are one of the most direct and effective ways to prevent the transmission of infections [61, 62]. It is likely that the high levels of awareness as evidenced by the enhanced knowledge scores for hand hygiene and PPE observed in our study are attributable to these regular training programs. The practical and immediate benefits of proper hand hygiene and PPE use (such as preventing the spread of infections) are well recognized by healthcare personnel. This recognition leads to stronger retention of knowledge [63]. Cheng et al*.* in their study suggest that healthcare personnel are more likely to remember and apply practices that are regularly performed and have an immediate impact on safety [64]. Although this observation is noteworthy, it is consistent with findings from a study in Uganda, which also highlighted a higher level of knowledge in these areas [29].

Overall, healthcare personnel demonstrated a strong understanding of nosocomial infections and their transmission. However, there was a noticeable lack of clarity regarding the time frame for when nosocomial infections typically manifest. Many healthcare personnel, including nearly 60% of nurses and 32.0% of doctors, were unclear about the 48 to 72-h period after which an infection is considered nosocomial. This lack of understanding is concerning, especially given that nurses and doctors are on the front lines of patient care and should be well-informed about infection timelines.

Laboratory technicians for instance had a better knowledge on the importance of hand hygiene than the other health care workers. This can be explained by the fact that they are more likely to be exposed to hand hygiene practices in the course of their work for example when handling specimens, equipment or when carrying out tests. This way, the consistent use of these measures not only enhances the knowledge but also increases the level of compliance [65]. However, the understanding of the participants was not specific on certain aspects of the hand hygiene for instance, the need to wash their hands when attending to patients with respiratory infections. This finding is consistent with a study by Ghalya and Ibrahim, which reported that while 89.6% of participants were knowledgeable about general hand hygiene practices, there were gaps in their understanding of certain key elements [43].

Regarding individual knowledge items related to PPE use, doctors exhibited superior knowledge compared to other healthcare personnel. However, a significant concern was that approximately one-third of participants incorrectly believed that PPE is only appropriate for laboratory and cleaning staff, which contrasts with a study where over 83.3% of participants correctly recognized that PPE is essential for all healthcare personnel, not just those in specific roles [43]. Additionally, more than half of the participants lacked thorough knowledge of proper PPE disposal mechanisms, with nearly 63.7% of nurses particularly lacking in this area.

Among the participants, the lowest degree of knowledge was noted with relation to sharp injury and disposal. This result is in line with research carried out in Saudi Arabia [66] and Uganda [29]. The greater emphasis on respiratory hygiene during and after the COVID – 19 pandemic era, when the study was done, may be one reason for this shortfall. Over 52.6% of participants demonstrated knowledge about the risks associated with recapping needles after use, aligning with a similar study in Nigeria, where most respondents recognized recapping as a risky practice that can expose healthcare personnel to occupational hazards [41]. According to Efstathiou et al*.,* recapped needles carry a significant risk of needle-stick injuries and should never be done. Consequently, needles ought to be disposed of immediately, without being taken out of the syringe [67].

A positive attitude towards infection prevention is a crucial pillar of effective infection control. In this study, approximately two-thirds of the participants (66.7%) demonstrated a positive attitude towards infection prevention. This finding aligns with similar studies in Saudi Arabia 61.5% [50], Ethiopia 63.8% [6], and 57.2% in another Ethiopian study [9]. However, it is lower compared to 78.6% in Nigeria [17], 76.4% [68], and 93.4% [69] in Ethiopia. Conversely, it surpasses the 46.7% reported in Trinidad and Tobago [38] and 40.8% in Ethiopia [4]. Notably, 44.9% of participants indicated that their ability to adhere to infection prevention standards is compromised by their workload. This contrasts with findings by Cimiotti et al*.,* who reported a higher prevalence of workload-related difficulties in applying infection prevention guidelines and established a link between staff numbers and hospital-acquired infections [70]. Therefore, addressing hospital staff fatigue could be an effective strategy to mitigate the spread of infections in healthcare settings.

It is concerning that 21.8% of participants indicated they would continue to report for duty despite exhibiting symptoms suggestive of a nosocomial infection, thereby potentially endangering both colleagues and patients. This proportion is notably higher than the 10% reported in a study conducted in South Africa, where participants indicated they would still attend work despite showing symptoms suggestive of COVID-19 [28]. This discrepancy suggests a potential gap in effectively communicating and enforcing appropriate health protocols among all healthcare personnel.

Over 90% of participants in a Saudi Arabian study felt that it is imperative to emphasize adequate hand cleanliness in medical curricula and healthcare settings, citing the considerable correlation between improper hand hygiene and patient morbidity and mortality [71]. Despite this consensus, our study found that 25.6% of participants frequently forgot to wash their hands. This lapse may be indicative of poor adherence to hand hygiene protocols, particularly since the facility is also an educational institution. Prior studies have demonstrated that the hand hygiene practices of students are significantly impacted by the habits of the role models they look up to, including nurses and doctors [72, 73].

### Limitations

Because of the cross-sectional form of the study, it is not possible to establish causal links between the variables under investigation. Furthermore, because semi-structured questionnaires were used, participant bias could have an impact on the results. Because conditions at other institutions can differ, the results’ generalizability may be limited by the small sample size and the particular institution’s focus. Even with a high response rate, recollection bias and social desirability can affect self-reported statistics.

## Conclusions

There are gaps in healthcare personnel’s knowledge and attitudes about infection prevention, as evidenced by the fact that 66.7% of research participants had a favourable attitude and 14.1% had good knowledge. The findings of an assessment of knowledge about PPE, hand hygiene, waste management, nosocomial infection time limitations, and sharps disposal revealed that some of them were not well understood. As such, there is a need for consistent and targeted training, increased staff to patient ratio and the presence of senior members of the staff demonstrating the right techniques. It is thus important that healthcare facilities implement and maintain these standards, incorporate infection control in training curricula, and ensure that PPE is adequately provided and used as recommended. Hence, there is the need for the constant assessment and feedback for the purpose of enhancement.

## Supplementary Information

Supplementary Material 1.

## Data Availability

The datasets used and analysed during the current study are available from the corresponding author on reasonable request.

## Abbreviations

COVID-19: Coronavirus disease 19
SARS-CoV-2: Severe acute respiratory syndrome coronavirus 2
WHO: World Health Organization
HAIs: Healthcare Associated Infections
HBV: Hepatitis B virus
HCV: Hepatitis C virus
HIV: Human immunodeficiency virus
PPE: Personal Protective Equipment
SPSS: Statistical package for social sciences
IPC: Infection prevention and control
