# Supplementary material for: Knowledge and attitude of nosocomial infection prevention and control precautions among healthcare personnel at Kiruddu Referral Hospital in Kampala, Uganda
Source: BMC Health Serv Res. 2025 Jan 28;25:161. doi: 10.1186/s12913-025-12219-5 (PMC11773981; doi:10.1186/s12913-025-12219-5)
Supplement: Supplementary file 1 — Supplementary Material 1. [file 12913_2025_12219_MOESM1_ESM.docx]

**Questionnaire for staff**

Dear Participant

Read the questions and mark your response off with a tick (√) in the circle provided and fill in the spaces with the answers.

**SECTION 1 - Socio – demographic characteristics of the staff**

**1. Gender**

Female

Male

**2. Age**

<20 years

21 – 29 years

30 – 39 years

40 – 49 years

>50 years

**3. Marital status**

Single

Married

Divorced/Separated

Widowed

**4. Working experience**

1 – 5

6 – 10

11 – 15

16 – 20

>20 years

**5. Occupation**

Doctor

Nurse

Physician

Other Specify; _________________________________________

**6. Educational level**

Secondary

Diploma

Bachelor’s Degree

Master’s Degree

PhD.

Other Specify; _________________________________________

**7. Duration of shift**

< 8hrs

8hrs

> 8hrs

**8. Mean number of patients interacted with during the shift**

< 5

5 – 10

11 – 15

16 – 20

> 20

**9. Have you got training on occupational health and safety?**

Yes

No

**10. Ever taken training on infection prevention and control**

Yes

No

**11. Duration of the training**

1 day

2 days

3 days

7 days

15 days

**Knowledge of the staff**

**General concept on nosocomial infection**

|  | **Agree** | **Not sure** | **Disagree** |
| --- | --- | --- | --- |
| Infections contracted at a hospital are known as nosocomial infections |  |  |  |
| An infection is nosocomial if it appears after;(48 – 72 hrs) |  |  |  |
| Medical equipment that is infected might spread nosocomial diseases |  |  |  |
| Nosocomial infection can be caused by bacteria only found in and around the hospital |  |  |  |
| Regardless of their diagnosis, any patient can spread infection |  |  |  |
| All bodily fluids aside from sweat should be considered potential infection sources |  |  |  |

**Hand hygiene**

|  | **Agree** | **Not sure** | **Disagree** |
| --- | --- | --- | --- |
| Practicing good hand cleanliness is the best defence against nosocomial infections |  |  |  |
| Those who have respiratory illnesses need to practice good hand hygiene |  |  |  |
| The danger of spreading hospital acquired germs is reduced by washing hands with soap and water |  |  |  |
| After taking off your gloves, you should wash your hands |  |  |  |
| Using an alcohol-based antiseptic for hand care is equally as effective as using soap if hands are not dirty |  |  |  |
| Putting on gloves makes washing your hands unnecessary |  |  |  |
| Before and after having direct patient contact, hand hygiene should be practised |  |  |  |
| The recommended minimum time for normal hand washing is between 40 and 60 seconds |  |  |  |

**Personal Protective Equipment (PPE) use**

|  | **Agree** | **Not sure** | **Disagree** |
| --- | --- | --- | --- |
| If there is no obvious contamination on the gloves, the same pair can be used for several patients |  |  |  |
| Protective barriers against infection are provided by PPEs such as masks and head coverings |  |  |  |
| The danger of developing nosocomial infections is eliminated by the use of PPEs |  |  |  |
| For their safety, PPEs are only appropriate for laboratory and cleaning employees |  |  |  |
| Only when there is blood contact should PPEs be worn |  |  |  |
| After being cleaned properly, gloves and masks can be used again |  |  |  |
| Old PPE should be disposed of in standard trash containers |  |  |  |
| Gloves should be changed when doing different procedures on the same patient |  |  |  |
| The most protective masks are those composed of cotton or gauze |  |  |  |
| If working with the same thing, masks and gloves can be reused |  |  |  |

**Sharp disposal and sharp injuries**

|  | **Agree** | **Not sure** | **Disagree** |
| --- | --- | --- | --- |
| To avoid injury, used needles should be recapped after use |  |  |  |
| After use, used needles should be twisted to prevent injury |  |  |  |
| When disposing of soiled sharps, shred them first |  |  |  |
| Sharps injuries ought to be treated without requiring reporting |  |  |  |
| In regular practice, needle-stick injuries are the least common |  |  |  |
| Injuries from an HIV-positive patient are managed with post-exposure prophylaxis |  |  |  |

**Waste management**

|  | **Agree** | **Not sure** | **Disagree** |
| --- | --- | --- | --- |
| Hospital waste has to be sorted before disposal |  |  |  |
| Cleaning and disinfection are the same |  |  |  |
| Hospital wards have to be cleaned only 2 times in 24 hrs |  |  |  |
| Waste at the hospital should be collected twice monthly |  |  |  |
| It is necessary to dispose of used PPE using the standard municipal disposal methods |  |  |  |

**Attitude of staff**

|  | **Agree** | **Not sure** | **Disagree** |
| --- | --- | --- | --- |
| Hand sanitizers irritate and make me feel dry. |  |  |  |
| Safety boxes should always be placed nearby locations where necessary operations are carried out. |  |  |  |
| Usually forget to wash my hands. |  |  |  |
| Sharps should always be disposed in sharps’ boxes |  |  |  |
| If I practise good hand hygiene am less likely to contaminate my patients |  |  |  |
| The importance of healthcare workers in preventing hospital acquired infections is crucial. |  |  |  |
| I would feel uncomfortable telling a healthcare worker to practise good hand hygiene. |  |  |  |
| I don’t change PPE between patients |  |  |  |
| Capacity to follow infection prevention standards is impacted by my workload. |  |  |  |
| Despite the discomfort, I would wear the necessary personal protective equipment. |  |  |  |
| Using alcohol-based hand sanitizers makes me feel safer than washing my hands with soap and water. |  |  |  |
| I will still report for duty even though I acquire an infection |  |  |  |
